# Challenges in diagnosing renal squamous cell carcinoma: a case report with emphasis on histopathology

**DOI:** 10.11604/pamj.2025.52.28.47915

**Published:** 2025-09-18

**Authors:** Prerna Tekulwar, Pravin Gadkari

**Affiliations:** 1 Department of Pathology, Jawaharlal Nehru Medical College, Datta Meghe Institute of Higher Education and Research, Sawangi (Meghe), Wardha, Maharashtra, India

**Keywords:** Squamous cell carcinoma, moderately differentiated, kidney, malignant squamous cells, case report

## Abstract

Squamous cell carcinoma (SCC) of the primary renal pelvis is an exceptionally rare tumor. It can often lead to infection, persistent discomfort, and nephrolithiasis. It is uncommon in the renal pelvis; instead, it usually occurs in the transitional epithelium of the urinary tract. Here, we report the case of a 60-year-old male who presented with a standing history of pain and tenderness over the right hypochondrium. A large, solid cystic lesion was noted in the right kidney during the imaging study. A moderately differentiated squamous cell carcinoma of the kidney was diagnosed on histopathology. A complete right nephrectomy was performed. Although the primary SCC of the kidney is rare, the uncommon location of this type of tumor emphasizes the case's relevance, and it should be taken into account when making a differential diagnosis of renal tumors.

## Introduction

Squamous cell carcinoma (SCC) in the primary renal pelvis is an incredibly uncommon tumor that makes up less than 1% of all kidney malignancies [1]. Clinically, the SCC presents in middle age; its incidence among males and females is almost equal. Renal stone-related SCC is more likely to occur when there is ongoing irritation, inflammation, and infection. Even though this type of kidney tumor is uncommon, it is important to consider a differential diagnosis when a renal mass coexists with a chronic renal stone [2]. Individuals may exhibit nonspecific signs and symptoms, and imaging tests may not be able to differentiate it from other kidney neoplasms or inflammatory diseases [3]. Occasionally, renal SCC may even present as xanthogranulomatous pyelonephritis (XGPN) [4]. Patients with renal squamous cell carcinoma often present at a later stage and are a challenge to diagnose at an early stage due to their rare radiological findings [5]. In patients receiving treatment for renal calculi, we emphasize the importance of renal biopsy because kidney squamous cell carcinoma cannot be detected in its early stages; therefore, the only way to diagnose it is through histopathological analysis, which can have serious consequences for the patients if missed [6]. Renal squamous cell carcinoma has a low survival rate due to its invasive nature and early metastasis; therefore, surgical resection is the treatment of choice if diagnosed in the early stage [7].

## Patient and observation

**Patient information:** a 60-year-old male came to the Outpatient Department of Surgery at Sawangi (Meghe) Wardha with the chief complaint of pain in the right hypochondrium region for 2 months, which was tender to touch and associated with hematuria. According to the patient, the pain was a pricking type. The patient had a history of hypertension. He had no history of prior surgery. Other medical history was noncontributory.

**Clinical findings:** on examination, percussion tenderness over the right kidney region was noticed. Laboratory investigations showed the serum urea and creatinine levels were 14 and 1 mg/dl. Urine examination revealed a 4+ hematuria and a 1+ proteinuria.

**Timeline of the current episode:** pain over the right hypochondrium for 2 months, gradually increasing and pricking in nature.

**Figure 1:**
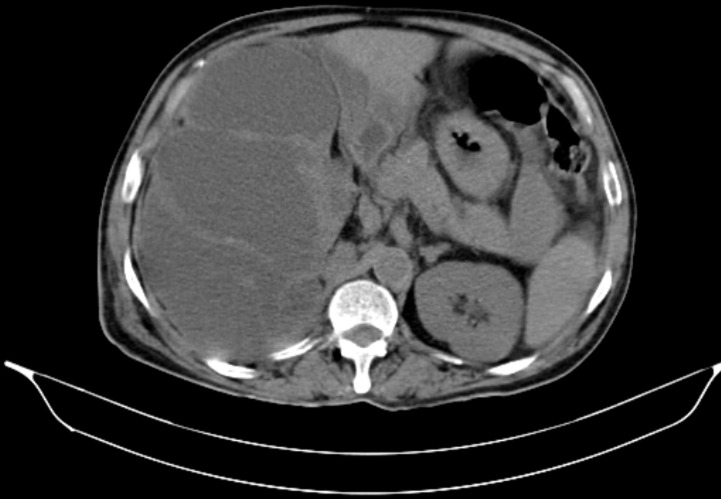
computed tomography abdomen-pelvis, large solid cystic lesion was noted in the right kidney

**Figure 2:**
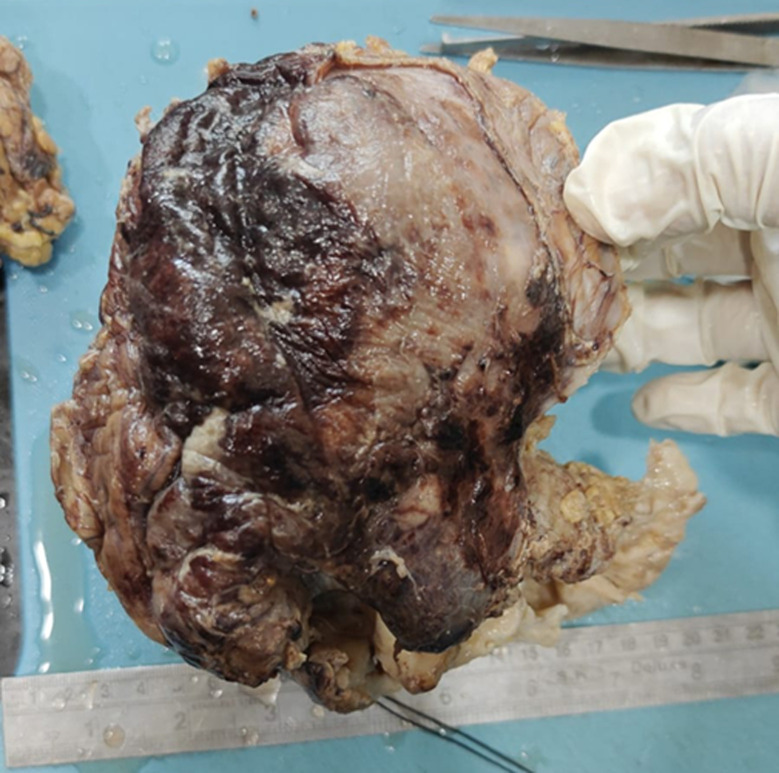
excised right nephrectomy specimen

**Figure 3:**
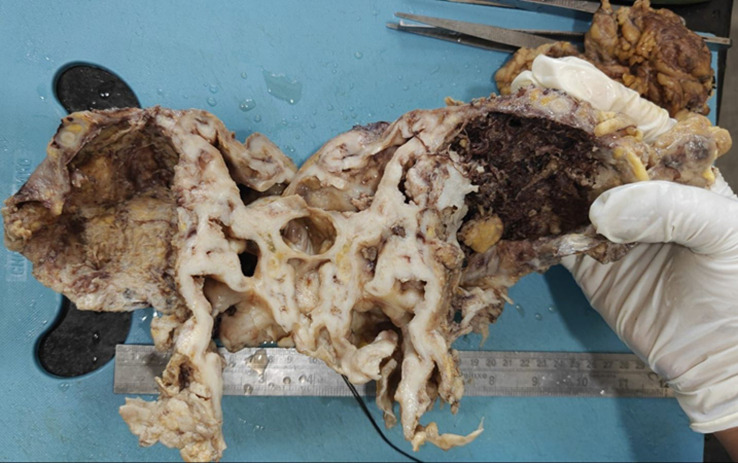
cut section of the tumor was white, solid, and homogenous with areas of hemorrhage and necrosis

**Figure 4:**
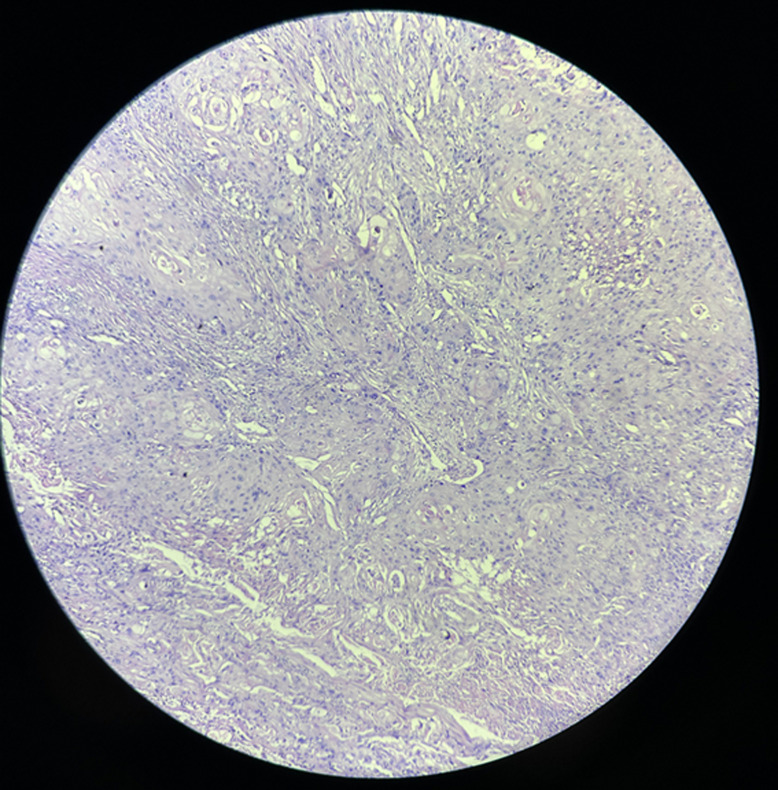
40X, high-power microscope, irregular infiltrating sheets of malignant squamous cells with destructive stromal invasion

**Diagnostic assessment:** a computed tomography of the abdomen-pelvis showed a large solid cystic lesion causing complete loss of the reniform architecture of the right kidney with multiple calculi (Figure 1). Following the report, the right nephrectomy was done, and the specimen was sent for histopathological diagnosis. Grossly, the excised right nephrectomy specimen was received. A single, unifocal, poorly circumscribed growth was identified involving the complete right kidney, measuring 15 x 15 x 7.5 cm (Figure 2). The cut section of the tumor was white, solid, and homogenous with areas of hemorrhage and necrosis (Figure 3). Under a 40X high-power microscope, irregular infiltrating sheets of malignant squamous cells with destructive stromal invasion were seen (Figure 4).

**Diagnosis:** histopathological findings confirmed the diagnosis of moderately differentiated squamous cell carcinoma of the right kidney.

**Therapeutic interventions:** the patient underwent right nephrectomy.

**Follow-up and outcome of interventions:** the patient had no metastases found, and he is receiving routine follow-up care.

**Patient perspective:** the course of therapy and the patient's level of recovery were both pleasing to the patient.

**Informed consent:** the patient granted a written, informed consent for the case report and any related images to be published.

## Discussion

Primary squamous cell carcinoma of the kidney is a rare cancer with a poor prognosis at the time of presentation [2]. About 5-10% of all urothelial tumors are derived from the urothelium of the renal pelvis and ureter, making them comparatively uncommon neoplasms. Eighty percent (80%) and 90% of them are urothelial carcinomas, and only five to seven percent of upper urinary tract malignancies are pure SCC [3]. The histological origin and mechanism for renal parenchyma SCC are still unknown; however, SCC of the renal pelvis was considered urothelial metaplasia [4]. Both males and females are typically present between the ages of 50 and 70. Predisposing variables include renal stones and persistent infections, which cause the squamous metaplasia and dysplasia, which in turn induce leukoplakia and neoplasia, which evolve into renal squamous cell carcinoma [6].

## Conclusion

Renal squamous cell carcinoma is always important to consider in a differential diagnosis when a renal mass coexists with a chronic renal stone. For patients with renal squamous cell carcinoma, early-stage diagnosis and surgical excision are the main goals of treatment. Additionally, this case report promotes comprehensive clinicopathological and radiographic correlation, coupled with a high index of suspicion and extensive differential diagnosis, necessary for these tumors to provide prompt, effective patient therapy.
